# Measuring the Potential Effects of Mirror Therapy Added to the Gold Standard Facial Neuromuscular Retraining in Patients With Chronic Peripheral Facial Palsy: Protocol for a Randomized Controlled Trial

**DOI:** 10.2196/47709

**Published:** 2023-07-07

**Authors:** Frédéric Dagenais, Catriona Neville, Liesbet Desmet, Sarah Martineau

**Affiliations:** 1 Faculté de Médecine Université Laval Québec, QC Canada; 2 Queen Victoria Hospital NHS Foundation Trust East Grinstead, West Sussex United Kingdom; 3 Department of Health and Care Artevelde University of Applied Sciences Gent Belgium; 4 European Institute for Otorhinolaryngology - Head & Neck Surgery Sint-Augustinus GZA Hospital Antwerp Belgium; 5 Centre de Recherche de l'Hôpital Maisonneuve-Rosemont École d’Orthophonie et d’Audiologie Université de Montréal Montreal, QC Canada

**Keywords:** facial rehabilitation, neuromuscular retraining, mirror therapy, facial paralysis, synkinesis, palsy, rehabilitation, neuromuscular, paralysis, palsies, physical therapy, physiotherapy

## Abstract

**Background:**

Facial neuromuscular retraining (fNMR) is a noninvasive physical therapy widely used to treat peripheral facial palsies. It consists of different intervention methods that aim to reduce the debilitating sequelae of the disease. Recently, the use of mirror therapy in the acute facial palsy and postsurgical rehabilitation contexts has shown promising results, suggesting its use as an adjunct to fNMR in treating patients with later stages of paralysis, such as the paretic, early, or chronic synkinetic.

**Objective:**

The main aim of this study is to compare the efficacy of an added mirror therapy component with fNMR in patients with peripheral facial palsy (PFP) sequelae in 3 different stages. The specific objectives of this study are to measure the effects of combined therapy compared to fNMR alone on (1) participants’ facial symmetry and synkinesis, (2) quality of life and psychological aspects of the participants, (3) motivation and treatment adherence, and (4) different stages of facial palsies.

**Methods:**

This study is a randomized controlled trial that compares the effect of fNMR combined with mirror therapy (experimental group: n=45) with fNMR alone (control group: n=45) in 90 patients with peripheral facial palsy presenting with sequelae 3-12 months after onset. Both groups will receive 6 months of rehabilitation training. Facial symmetry and synkinesis; participants’ quality of life; and their psychological factors, motivation, and compliance will be assessed at baseline (T0), 3 months (T1), 6 months (T2), and 12 months (T3) postintervention. Outcome measures are (1) changes in facial symmetry and synkinesis assessed with facial grading tools, (2) quality of life changes with patient questionnaires, and (3) therapy motivation with a standardized scale, as well as adherence to treatment with metadata. Changes in facial symmetry and synkinesis will be judged by 3 assessors blinded to group assignment. Mixed models and Kruskal-Wallis, chi-square, and multilevel analyses will be conducted according to the appropriate variable type.

**Results:**

Inclusion will start in 2024 and is anticipated to be completed in 2027. The 12-month follow-up will be completed with the last patient in 2028. We expect patients included in this study to experience improvement in facial symmetry, synkinesis, and quality of life, regardless of group assignments. A potential benefit of mirror therapy for facial symmetry and synkinesis could be noted for patients in the paretic phase. We hypothesize better motivation and adherence to treatment for the mirror therapy group.

**Conclusions:**

The results of this trial may provide new guidelines for PFP rehabilitation with patients dealing with long-term sequelae. It also fills the need for robust evidence-based data in behavioral facial rehabilitation.

**International Registered Report Identifier (IRRID):**

PRR1-10.2196/47709

## Introduction

### Background

Peripheral facial palsy (PFP) with sequelae is a debilitating condition that can significantly impact a patient’s quality of life due to functional, social, and psychological factors [1]. Facial asymmetry and the inability to express emotions and smile adequately can make communication challenging, leading to high levels of social problems, frustration, anxiety, and depression [1]. In nonflaccid facial palsy, patients may have hyperkinesis, muscle contracture, and synkinesis, which can affect normal activities such as drinking, eating, and articulating and can cause embarrassment and, in some cases, facial pain [2,3]. Therefore, providing adequate interventions to help patients deal with these issues is essential. Physical therapy is a type of nonsurgical treatment shown to help manage the sequelae previously mentioned in patients with nonflaccid chronic peripheral facial palsy [4]. Optimal facial rehabilitation modalities could also differ depending on the stage of nonflaccid PFP [5]. Although there are no generally accepted guidelines for the different stages, some authors define the paretic phase as recovery from 3 to 6 months after onset, while the early synkinetic phase would be defined as 6 to 9 months after onset [5,6]. The chronic synkinetic phase would begin at 9 months after onset, as defined by Beurskens et al [7].

### Facial Neuromuscular Retraining

Mime therapy [8,9] and facial neuromuscular retraining (fNMR) [10] are 2 rehabilitation techniques for managing facial palsy sequelae that stand out in the literature [11]. Mime therapy consists of facial massage and specific facial retraining to coordinate both hemifaces by using emotional cues to express a particular facial movement or by starting to use a specific muscle to create an expression [8]. Similarly, fNMR consists of relearning motor patterns to improve facial movements through conscious, consistent, and slow selective activation of facial muscles using individualized training programs made by qualified facial therapists [12]. This therapy aims to normalize facial muscle functions by inhibiting synkinetic contractions and improving resting tone altered by contractures [13]. In the literature, these 2 therapies have recently merged into one called fNMR, which combines techniques such as soft tissue massage, biofeedback, education, relaxation, and selective movement retraining [14]. fNMR is considered a well-supported approach that has been studied in many cohort studies [14].

### Mirror Therapy in Facial Rehabilitation

**Figure 1:**
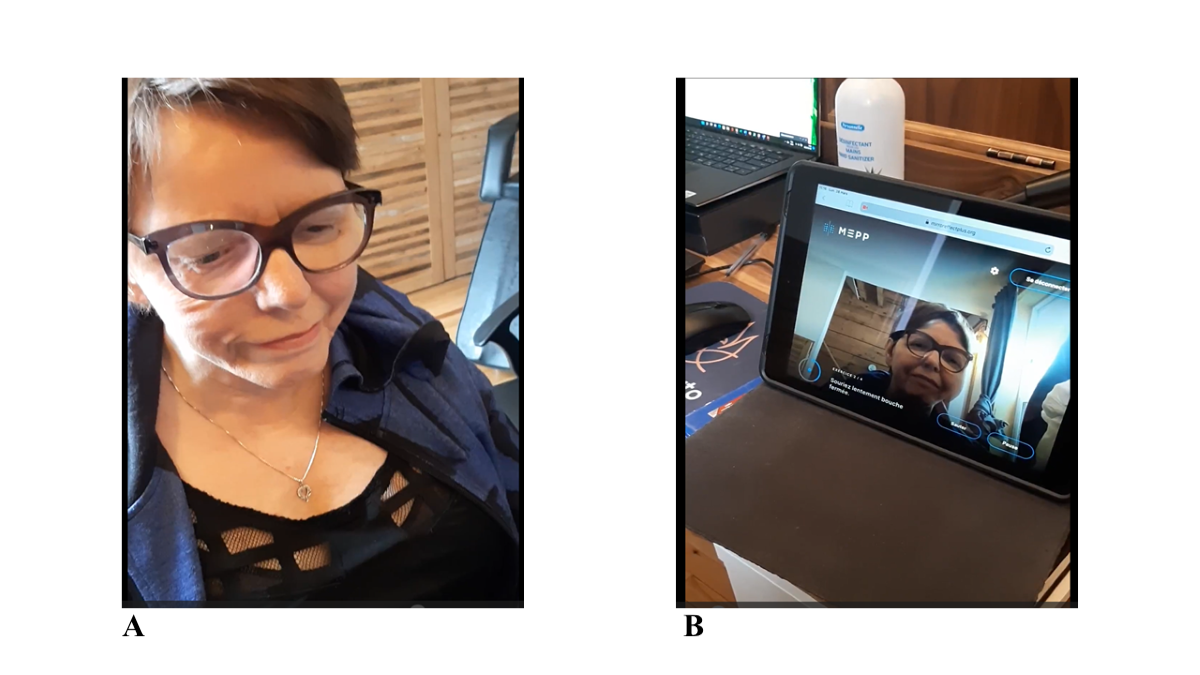
Representation of the setting with mirror therapy for facial rehabilitation. (A) Patient sits in front of the computer with a webcam. (B) Visual biofeedback of modified face.

Mirror therapy is a form of motor therapy that involves placing a mirror in the patient’s midsagittal plane, reflecting the unaffected limb or body part onto the affected one, creating the illusion of normal movements on the absent or affected side [15]. It is based on the brain’s ability to undergo cortical reorganization [15]. The first clinically reported effects of mirror therapy were pain reduction in arm amputees and alleviation of hemiparesis [16]. Since then, this therapy has been used in other contexts, including facial rehabilitation and uses specific computer software to replicate the unaffected side over the affected side [17] (Figure 1).

A recent study investigated the effects of mirror therapy over 3 months on 32 patients diagnosed with facial nerve palsy less than 12 months after acoustic neuroma resection surgery [17]. The control group received traditional rehabilitation with mime therapy, while the experimental group had to perform an additional weekly session of mirror therapy using specific software and motor imagery exercises with different emotions or gestures. Both groups were evaluated for facial symmetry using the House-Brackmann Facial Nerve Grading system [18], Sunnybrook [19], and Facial Clinimetric Evaluation (FACE) [20]. Although every subject demonstrated improvement in facial symmetry, the experimental group showed significantly better results than the control group for House-Brackmann scores and quality of life. In a recent case report, mirror therapy and motor imagery were used with a small group of patients (n=5) with chronic facial palsy to reduce synkinesis, which resulted in the reduced muscular activity of synkinetic muscles with mirror therapy [21].

A recent longitudinal study recruited patients with Bell palsy within 14 days of onset and followed them for 1 year [22,23]. The control group received only basic counseling in their rehabilitation, while the experimental group followed a new protocol called the Mirror Effect Plus Protocol (MEPP), which was mainly based on mirror therapy [24] and motor imagery [25]. One year after onset, significant results included less synkinesis development, increased facial symmetry, and improved self-reported quality of life in the experimental group [23]. Although the MEPP shows interesting results in patients with Bell palsy when initiated in the acute phase, to our knowledge, there is very limited evidence for mirror therapy as an effective treatment tool for patients presenting with long-standing peripheral facial paralysis from different etiologies.

### Expected Effects of Rehabilitation Techniques

Based on the work of Diels and Beurskens [14], fNMR is expected to improve facial symmetry and reduce synkinesis in long-standing facial palsy. Increased resting tone on the patient’s affected side often creates muscle tightness or contractures, leading to decreased range of motion, alteration of facial expressions, and patient discomfort. Therefore, soft tissue mobilization and specific facial manipulations can reduce such tightness and increase facial symmetry at rest [14]. To inhibit synkinesis, the therapy seeks to recognize abnormal movement patterns and uses different strategies to reduce them while activating desired facial muscles [5]. Patients must be instructed to perform slow, small, and controlled facial expressions or movements. As the patient’s control improves and the synkinesis movements remain suppressed, the range of motion, facial function, and symmetry are believed to improve [14]. As proprioceptive input is absent in facial muscles, fNMR uses different education and biofeedback strategies, such as surface electromyography and touch, to help patients focus on the right muscle contraction. Although successful results have been demonstrated in the past with patients in the chronic phase, more randomized controlled trials are required to assess its efficacy [4]. It has been noted that when traditional mirrors are used in practice sessions as biofeedback, the mismatch between the motor command and the visual feedback associated with the affected side might result in maladaptive muscle hyperactivity and compensation, which is counterproductive with fNMR [26,27]. In this context, adding mirror therapy during practice is an interesting idea because it might avoid such discrepancies between motor command and sensory input [23]. However, the potential added value of mirror therapy for long-standing facial palsies remains to be proven.

### Study Aims

There is a growing interest in exploring the effect of adding mirror therapy as a biofeedback tool in fNMR, particularly in patients with peripheral facial palsy dealing with sequelae 3-12 months after onset. Therefore, the general aim of this protocol is to compare the potential effects of adjunctive mirror therapy with the gold standard fNMR on facial function and rehabilitation in nonflaccid facial paralysis. The specific objectives of this study are (1) to measure the effects of combined therapy compared to fNMR alone on participants’ facial symmetry and facial synkinesis, (2) to measure the effects of combined therapy versus fNMR alone on the quality of life and psychological aspects of the participants, and (3) to measure the effects of combined therapy versus fNMR alone on motivation and treatment adherence.

## Methods

### Eligibility Criteria

To be included in the study, patients must be 18 years or older, experiencing their first PFP, be from 3 months to 12 months after onset, and must have a score 4 or 5 on Facial Nerve Grading System 2.0 (FNGS 2.0) [28]. In addition, they must have sufficient cognitive status, measured by a cutoff score of 23 on the Montreal Cognitive Assessment [29,30], and present with no active mental health diagnosis. They should also not present with a history of neurologic diseases affecting facial function. Patients with concomitant therapies or botulinum toxin type A injections, presenting with bilateral palsies or with medically uncontrolled metabolic diseases, will be excluded from the study. Access to an internet connection and basic computer literacy will also be verified as eligibility criteria.

### Recruitment and Compensation

As determined, thanks to a power calculation measure based on Martineau et al [22] to reach a power of 0.8 with a size effect of 0.6 and an α of .005, 90 patients with peripheral facial palsy of diverse etiologies in the paretic (n=30), early synkinetic (n=30), or chronic synkinetic phase (n=30) will be recruited to the study. They will then be randomized into 2 groups, mirror therapy plus fNMR or fNMR alone, by computerized block randomization. No compensation or incentive is planned to be provided at this time. Recruitment is planned to be conducted in 3 tertiary care centers (hospitals) in Canada, England, and Belgium (Hôpital Maisonneuve-Rosemont, Queen Victoria Hospital, and Sint-Augustinus GZA Hospital, respectively). A telehealth option will be available for the participants via institutionally secured platforms.

### Study Procedures

**Figure 2:**
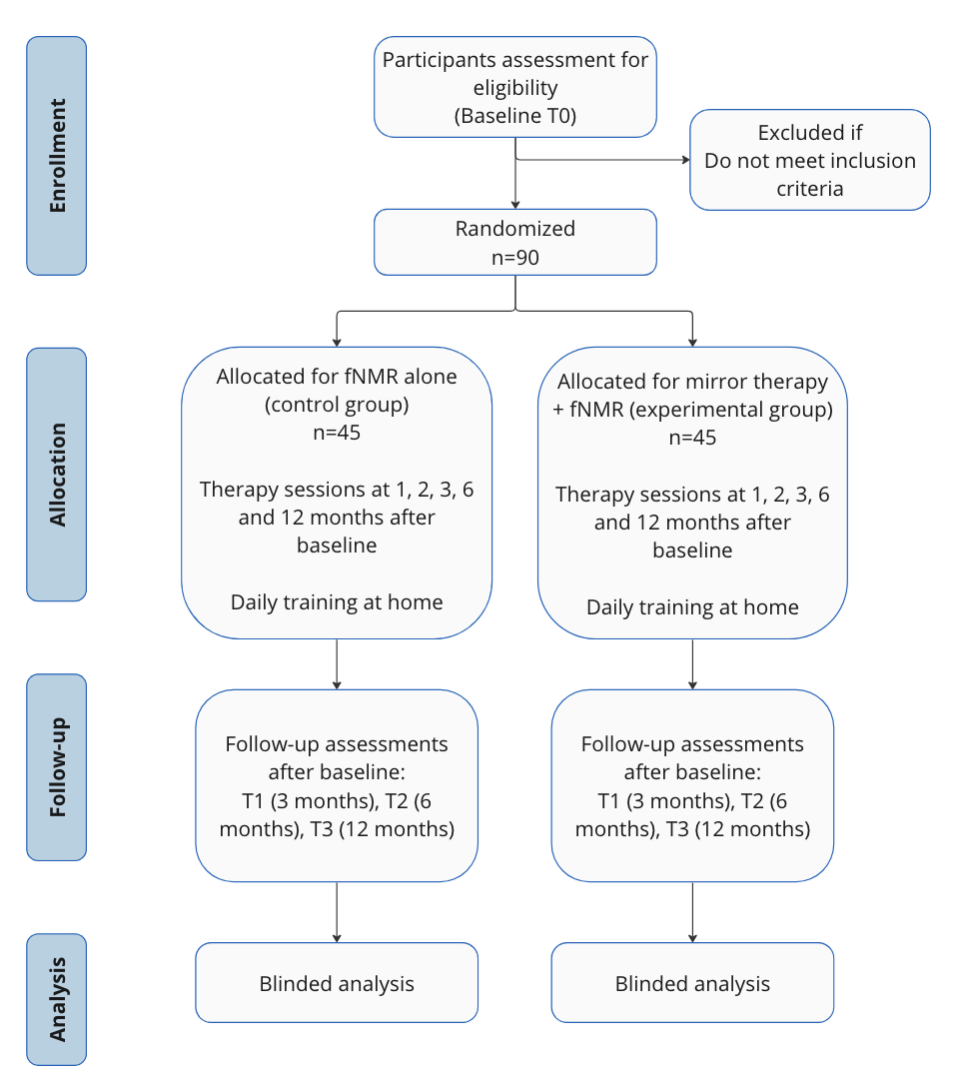
Overall study design. Participants in the control group and in the experimental group are assessed at baseline (T0), 3 months postintervention (T1), 6 months postintervention (T2), and 12 months postintervention (T3). fNMR: facial neuromuscular retraining.

This trial will compare the effect of mirror therapy in addition to gold standard fNMR (experimental group) over fNMR alone (control group) in patients with peripheral facial palsy dealing with sequelae 3-12 months after onset. During 6 therapy sessions (Textbox 1), all patients will benefit from standardized education on facial anatomy and physiology, eye care, and management of facial dysfunction (eg, eating, drinking, speaking, and nonverbal communication), as well as from rehabilitation to increase symmetry and decrease synkinesis, specific to their needs. These sessions will be provided by speech-language, physical, or occupational therapists, all certified by their respective professional corporation. In addition, a minimum of 5 years of expertise in facial palsy training is required to be included in the study. These clinicians will also perform the different study measurements, but 3 judges (also experts in facial therapy but blinded to therapy allocation) will then analyze the data. The mirror therapy group will also receive modified visual feedback during facial retraining, along with appropriate instructions about its use. In parallel, patients will be prescribed daily specific at-home rehabilitation depending on the nature of their group. Patients in the combined group (mirror therapy plus fNMR) will do their therapy at home with the MEPP 2.0 app [31], while patients in the fNMR group alone will do their therapy at home with the Physitrack app [32]. Figure 2 presents the overall study design.

**Frequency and length of appointment.**

- Sessions
  Session 1 (1 hour to 1 hour and 30 minutes) for initial assessment and start of management.
  Session 2 (1 hour) 1 month after session 1.
  Session 3 (1 hour) 1 month after session 2.
  Session 4 (1 hour) generally 1-2 months after session 3.
  Session 5 (1 hour) is generally 3 months after session 4.
  Session 6 (1 hour) final session generally 6 months after session 5, then discharge to continue independently.
- The total treatment course will last 12 months.
- 2-3 sessions of 10 to 15 minutes of daily home rehabilitation are expected to be done by the patients from baseline until 12 months.

### Measures

For data collection, each measure will be taken at baseline, 3 months, 6 months, and 1-year postintervention. Evaluation sessions will be recorded, allowing 3 independent evaluators to blindly assess the objective outcome measures. The final assessment (T3) will measure the training retention of participants following therapy sessions.

### Facial Function Assessment Tools: Symmetry and Synkinesis

Different tools will be used to measure the effects of combined therapy compared to fNMR alone on facial symmetry and synkinesis of the participants. First, Emotrics [33], a designed open-source machine learning algorithm, will enable objective facial metrics for patients with facial paralysis. The algorithm uploads a front-view picture of the patient’s face into the program. Then, it automatically places 68 facial landmarks and computes a set of facial metrics relevant to facial palsies, such as palpebral fissure width, smile excursion, and smile and brow symmetry. Second, the FNGS 2.0 [28] and Sunnybrook Facial Grading Scale [19] will be used by 3 independent blinded evaluators to assess facial symmetry at rest, voluntary facial movement, and synkinesis. The FNGS 2.0, also called the modified House-Brackmann scale, is a scale where 4 regions of the face are assessed and scored into different categories depending on the degree of movements and synkinesis presence [34]. It generates a final score between 4 and 24 and converts it into one of the original House-Brackmann categories ranging from I (normal function) to VI (total paralysis). In other words, it incorporates synkinesis scoring and regional scoring to the original validated House-Brackmann scale [28]. As described by Vrabec et al [28], this revised scale presents high inter- and intraobserver agreement with the original scale while providing additional information. On the other hand, the Sunnybrook Facial Grading Scale uses a continuous scale of 0% (total facial paralysis) to 100% (normal facial function and symmetry). This scale has been chosen because of its high sensitivity to changes induced by the intervention [34].

### Patient-Related Outcome Measures: Quality of Life

To measure the effects of combined therapy compared to fNMR alone on the quality of life and psychological issues of the participants, different patient-related outcome measures will be used including the FACE [20] and the Facial Disability Index (FDI) [6]. The FACE assesses 6 main domains: facial comfort, facial movement, oral function, lacrimal control, eye comfort, and social function through 15 questions. Overall, facial function depends on a score previously calculated that ranges from 0 (worst) to 100 (best) [20]. It has been previously demonstrated that FACE effectively discriminates the normal functional state of a person who does not have facial palsy from the state of a person have the disease [20]. Furthermore, the FACE can be used in different clinical contexts, such as assessing the evolution of patients’ facial palsy handicaps from distinct etiologies and reporting changes in patients’ perceptions of social and functional plans [20]. Kahn et al [20] performed an exhaustive process to validate the instrument, which is internationally used for its validity, sensitivity, specificity, and reliability [35]. On the other hand, the FDI has 10 questions that assess 2 domains: physical and social function. Social function scores from 0 (worst) to 100 (best), while physical function scores from −25 (worst) to 100 (best) [6]. Its validity and reliability have been demonstrated by VanSwearingen and Brach [6]. While the FACE scale provides more domains, it lacks measures about specific functional information, such as speech, which is why the use of FDI could be complementary [36].

### Motivation and Adherence to Treatment

**Figure 3:**
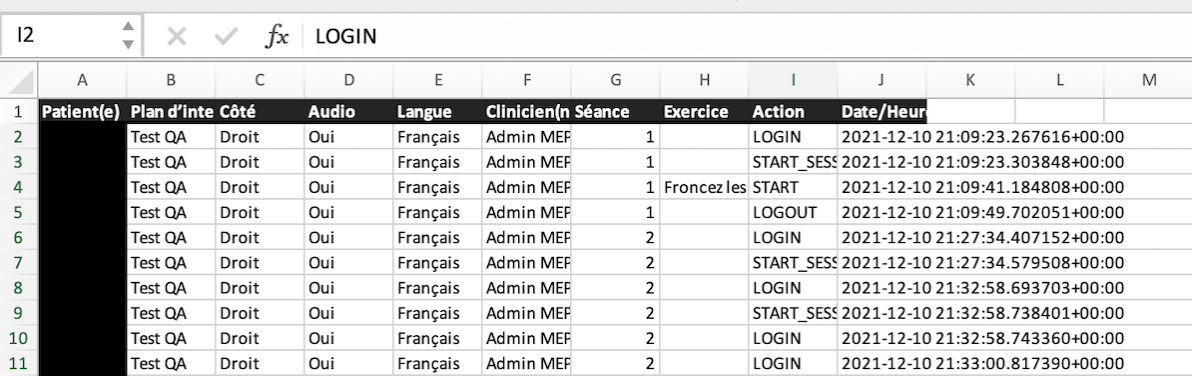
Example of metadata about compliance with the Mirror Effect Plus Protocol website.

To measure the effects of combined therapy compared to fNMR alone on the rehabilitation process of the participants and motivation to treatment, the intrinsic motivation inventory [37] will be used. This multidimensional tool has been developed to assess participants’ intrinsic motivation toward specific activities in laboratory experiments. Interest or enjoyment, perceived competence, effort, pressure or tension, usefulness, perceived choice, and relatedness of a task are all assessed while the participant performs a specific task. The scale has been validated in several contexts [37,38]. Patients’ compliance to treatment will be assessed through intrinsic features of both websites that record metadata every time the participant logs in. These features allow therapists to see if the patient completed their rehabilitation program as recommended. Figure 3 shows an example of the metadata that the MEPP website can generate on an Excel (Microsoft Corp) sheet about compliance with treatment.

### Statistical Analysis

As mentioned, a power calculation was done based on a previous study [23] for the primary outcome (changes from baseline to 6 months). Every measure of facial symmetry and synkinesis will be judged by 3 assessors, blinded to group assignment. Intraclass correlation coefficients will be calculated to ensure interrater reliability, and the resulting means of the assessors’ findings will be used for further statistical analysis. We will first gather descriptive statistics to provide summary data. Then, for continuous variables (Emotrics measurements, Sunnybrook global score, FNGS 2.0 global scores, FACE score, FDI, and intrinsic motivation inventory), a mixed model analysis with patients as random variables and time+group assignment as fixed variables will be considered, with auto-regressive covariance structure, given the structure of the time points for the assessments. Comparison between the timing of recruitment after PFP onset (3-6 months after onset vs 6-9 months vs 9-12 months) and outcomes at T2 and T3 will be specifically measured using multilevel analysis. Kruskal-Wallis analyses will be considered for ordinal variables (synkinesis subscores, symmetry subscores, and motivation subscores). Finally, chi-square analysis will be considered for dichotomous variables (adherence yes or no). Data will be encoded with Excel sheets, and SPSS (IBM Corp) will probably be used for statistical analysis.

### Ethical Considerations

Ethics approval will be sought by January 2024 for an international and multicentric randomized control trial involving tertiary care centers in Canada, the United Kingdom, and Belgium. Only participants who are adults and able to consent will be recruited for this study. This study presents minimal risks for participants, as only behavioral therapy will be provided in the study procedures: there will be no use of approved or unapproved drugs, biologics, food, or dietary supplements. This study does not evaluate the safety or effectiveness of a device. No protected health information will be collected or placed into electronic medical records. No compensation or incentive is planned to be provided at this time.

### Data Management Plan

All data will be collected in an electronic format. In the unanticipated event that data cannot be collected electronically, the same information will be collected via pen and paper, transferred to Excel, and the paper format will be destroyed at the earliest opportunity. All collected data will be strictly confidential, within the limits established by law, and a numerical code will identify each participant. Data will be recorded in the cloud of the University of Montreal, specially developed for research purposes, and only the team members will have access. Furthermore, all data will be conserved, for a minimum period of 7 years, after which they will be destroyed. None of the potential scientific publications or communications related to the study will contain participant-identifiable information. The researchers and the research assistants will only consult data. No facial recordings or sensitive or medical information will be held on the websites used in the study. Only the name, email address, and information about the affected side of the facial palsy (for duplication of the unaffected side purpose) and preference about sound level (with or without sound) will appear. Moreover, both websites use the latest versions of tools and information components, such as long time support, which offer support and security updates regularly, and a specialized technical team will ensure security updates and support for maximal security.

## Results

The inclusion will start in late 2024, and data collection is anticipated to be completed over 3 years by 2027. Therefore, the last patient will have their final follow-up (T3) in 2028. Data analysis will be completed in 2028-2029. The results will be published in 2030.

## Discussion

### Main Study Findings

The primary objective of this protocol is to compare the potential effects of adjunctive mirror therapy with the gold standard fNMR for facial function and rehabilitation in cases of nonflaccid facial paralysis. In particular, this study aims to evaluate the impact of combined therapy versus fNMR alone on participants’ facial symmetry, facial synkinesis, quality of life, motivation, and treatment adherence.

### Comparison With Prior Work and Scientific Value of This Work

A recent Delphi study has confirmed the significance of fNMR and mime therapy in treating patients with various types of peripheral facial paralysis [39]. While mirror therapy was not unanimously agreed upon for inclusion at that time, subsequent data published after the study indicated that mirror therapy, when initiated a few days after the onset (10-14 days) of Bell palsy, had notable benefits. These benefits included a significant improvement in facial symmetry, quality of life, and a reduction in the development of synkinesis [23]. Similar positive outcomes have also been observed in patients with facial palsy after acoustic neuroma resection and in those who underwent lengthening temporalis myoplasty surgery [17,24]. However, despite these findings, there is a dearth of research examining the potential effects of mirror therapy in patients with peripheral facial palsy experiencing delayed recovery and sequelae at least 3 months after the onset, compared to more traditional therapies like fNMR.

Irrespective of the etiology and group allocation (fNMR vs mirror therapy), our hypothesis posits that all patients with peripheral facial palsy enrolled in this study will experience improvements in facial symmetry, synkinesis, and quality of life [11,23,40]. Drawing on previous studies [23,41] and considering that mirror therapy appears most beneficial when initiated early, we anticipate mirror therapy to have greater benefits for patients in the paretic phase (3-6 months after onset), particularly in terms of restoring facial symmetry. Patients presenting with synkinesis at the time of recruitment should experience a decrease in synkinesis in the fNMR group, as indicated by several previous studies [4,5,7,13,14]. Similarly, a decrease in synkinesis is expected in the mirror therapy group, but no significant differences between the 2 groups are anticipated. Moreover, in line with previous findings [5,23], we expect both the fNMR and mirror therapy groups to demonstrate improvements in self-reported quality-of-life measures. Additionally, motivation is crucial in achieving positive therapy outcomes [42] and will be specifically assessed in this study. The hypothesis of greater motivation with mirror therapy is based on a previous study where patients reported that performing facial exercises while viewing a symmetrical face aided them as it represented their desired goal [41]. On the contrary, some patients may experience emotional responses when confronted with a face they perceive as “lost” due to facial paralysis.

Finally, we anticipate that patients in the mirror therapy group will exhibit better treatment adherence compared to those undergoing fNMR alone. Adherence to facial retraining has been shown to be influenced by various factors, including time constraints due to other daily activities and accessibility to the training facilities [43]. Facial therapists often overestimate their patients’ commitment to the facial rehabilitation process, with data indicating that only 30% of patients fully comply [43]. Nonadherence to therapy can impact treatment outcomes, highlighting the importance of measuring adherence in this study. Objective measures of adherence using metadata will be used, providing strength compared to relying solely on subjective measures of compliance. Subjective measures of compliance (such as patient questioning) may be influenced by patients’ memory or perceptions [44]. Furthermore, the postpandemic shift toward telehealth for service delivery may facilitate adherence [44]. In this study, we will document whether participants received care via telehealth and examine whether this factor appears to influence compliance.

### Strengths and Limitations

The primary strength of this protocol lies in its international multicentric collaboration, which will facilitate the examination of a substantial number of participants representing diverse etiologies of peripheral facial nerve palsy (n=90, with 45 patients per group). The randomized controlled trial design used in this study allows for assessing the potential additional benefits of incorporating mirror therapy into the rehabilitation of PFP. This investigation has the potential to contribute to an evidence-based approach for clinicians involved in the care of these patients.

However, the study design does have certain limitations. First, it is not feasible to blind both the patients and treating clinicians to the assigned conditions, which could introduce bias in terms of motivation and perception of treatment efficacy. To mitigate these risks, blinded judges will be involved in assessing participants, and therapists will use a standardized feedback approach in both therapy sessions and assessments. Additionally, using Emotrics will allow for more objective facial metrics at each assessment time point [33,45]. Second, the inclusion of paretic patients in the study poses a potential confounding factor due to the occurrence of spontaneous recovery in certain cases of facial palsy [3]. However, the large number of participants and their enrollment at least 3 months after the onset of symptoms should help mitigate potential biases. It is worth noting that in Bell palsy, the most common cause of PFP, approximately 70% of patients experience full recovery within 3 months of onset [3]. The advantage of including patients across such a broad timeframe (3-12 months after onset) is to recruit a cohort that is as representative as possible of the clinical reality of facial rehabilitation in different countries.

### Conclusions

By comparing treatment modalities such as fNMR and mirror therapy within the scope of this international study, clinicians can gain valuable insights into effective approaches for addressing specific facial sequelae in patients. Understanding which modalities may be more effective at different stages of PFP is important for the field. Moreover, this study has the potential to shed light on the impact of motivation and treatment adherence on treatment outcomes.

## Abbreviations

FACE: Facial Clinimetric Evaluation
FDI: Facial Disability Index
FNGS 2.0: Facial Nerve Grading System 2.0
fNMR: facial neuromuscular retraining
MEPP: Mirror Effect Plus Protocol
PFP: peripheral facial palsy

## Data Availability

Data sets gathered in this study are planned to be deposited in the publicly available repository of Montreal University (Borealis Data).
